# 29043 Using Milestones to Judge the Progress of Clinical Informatics Fellows Compared with their Personal Goals

**DOI:** 10.1017/cts.2021.561

**Published:** 2021-03-30

**Authors:** Douglas S. Bell, Kevin Baldwin, Eric Cheng, Michael Pfeffer

**Affiliations:** UCLA Clinical Informatics Fellowship Program, and UCLA Clinical & Translational Science Institute, Los Angeles, CA

## Abstract

ABSTRACT IMPACT: We report a novel metric for assessing clinical informatics fellows relative to their personal goals, using standardized milestones that have been approved for the field by ACGME. OBJECTIVES/GOALS: ACGME has defined 20 milestones that serve as the goals for fellows in clinical informatics. Each fellow is rated from 1 to 5 on the achievement of each milestone, where 1 is entry-level, 4 is the level expected of a graduating fellow, and 5 is aspirational. We assessed fellows’ progress toward the personal goal levels that they set for each milestone. METHODS/STUDY POPULATION: At the start of the fellowship, we asked each fellow to rate the personal target levels that they want to achieve for each milestone. Since the default target level of achievement for a graduating fellow is a 4, we asked fellows to document exceptions from this target. We calculated a metric for each fellow’s achievement of each milestone as their achievement rating (assigned by mentors and rotation leaders during the semi-annual Clinical Competency Committee meeting) divided by the fellow’s desired level of achievement. In summarizing across the milestones, we counted those milestones having achievement metrics >=1.0 as ‘achieved,’ and then for milestones that were not achieved, we calculated an average for the fellow. RESULTS/ANTICIPATED RESULTS: As of June, 2020, our two graduating 2nd-year fellows had fully met 9/20 and 18/20 milestones, respectively. For the unmet milestones they averaged 81% and 85% achievement. The largest shortfalls were 75% achievements in Assessing User Needs for one fellow, and in Recognition of Errors for the other. One of our three 1st-year fellows had fully met 3/20 milestones; the other two had met none at 1st-year’s end. For unmet milestones, the 1st-year fellows’ average achievement metrics were 69%, 67%, and 52%. The greatest shortfalls were in Resource Utilization (creating job descriptions, budgeting etc.) and in Communication with Patients and Families. However, the rotations that would expose them to project management and to patient-facing systems such as MyChart come in our 2nd-year. DISCUSSION/SIGNIFICANCE OF FINDINGS: Assessing milestones met plus the percent achievement for those not yet met provides a useful metric for comparing fellows and identifying areas in need of more training. Although milestones will soon change to reflect the recent practice analysis for clinical informatics, we expect that this approach to assessing fellows will remain equally useful.

